# Prognostic Factors of Peritoneal Metastases from Colorectal Cancer following Cytoreductive Surgery and Perioperative Chemotherapy

**DOI:** 10.1155/2013/978394

**Published:** 2013-04-18

**Authors:** Yutaka Yonemura, Emel Canbay, Haruaki Ishibashi

**Affiliations:** ^1^NPO to Support Peritoneal Surface Malignancy Treatment Unit, Peritoneal Carcinomatosis Center, Kishiwada Tokushukai and Kusatsu General Hospital, 1-26 Haruki-Moto-Machi, Kishiwada City, Osaka 596-0032, Japan; ^2^Kishiwada Tokushukai Hospital, Osaka, Kishiwada 596-8522, Japan

## Abstract

*Background*. Prolonged survival of patients affected by peritoneal metastasis (PM) of colorectal origin treated with complete cytoreduction followed by intraoperative hyperthermic intraperitoneal chemotherapy (HIPEC) has been reported. However, two-thirds of the patients after complete cytoreduction and perioperative chemotherapy (POC) develop recurrence. This study is to analyze the prognostic factors of PM from colorectal cancer following the treatment with cytoreductive surgery (CRS) + POC. *Patients and Methods*. During the last 8 years, 142 patients with PM of colorectal origin have been treated with CRS and perioperative chemotherapy. The surgical resections consisted of a combination of peritonectomy procedures. *Results*. Complete cytoreduction (CCR-0) was achieved at a higher rate in patients with peritoneal cancer index (PCI) score less than 10 (94.7%, 71/75) than those of PCI score above 11 (40.2%, 37/67). Regarding the PCI of small bowel (SB-PCI), 89 of 94 (91.5%) patients with ≤2 and 22 of 48 (45.8%) patients with SB-PCI ≥ 3 received CCR-0 resection (*P* < 0.001). Postoperative Grade 3 and Grade 4 complications occurred in 11 (7.7%) and 14 (9.9%). The overall operative mortality rate was 0.7% (1/142). Cox hazard model showed that CCR-0, SB-PCI ≤ 2, differentiated carcinoma, and PCI ≤ 10 were the independent favorite prognostic factors. *Conclusions*. Complete cytoreduction, PCI, SB-PCI threshold, and histologic type were the independent prognostic factors.

## 1. Introduction

In the past, peritoneal metastases (PM) from colorectal cancer have been considered a terminal stage of disease, and patients were offered the best supportive care and/or systemic chemotherapy with or without palliative surgery. Surgery or chemotherapy alone did not improve the patients' survival and results in a median survival of 5–7 months [1, 2]. Over the past two decades, a new therapeutic alternative approach based on the combination of surgery with chemotherapy was developed as a treatment of PM. In this curative intent, the macroscopic disease was treated with cytoreductive surgery (CRS) followed by treating residual microscopic disease with an intraoperative hyperthermic intraperitoneal chemotherapy (HIPEC) and/or early postoperative intraperitoneal chemotherapy (EPIC). 

In 2003, prolonged survival of patients affected by PM of colorectal origin with complete cytoreduction followed by HIPEC was reported in a prospective randomized phase III trial [3]. Furthermore, Sugarbaker's peritoneal cancer index (PCI) [4], histopathological type, and postoperative systemic chemotherapy were additional significant prognostic factors [3]. 

Beside these improvements, the long-term outcome of these patients is still dismal, and approximately two-thirds of the patients who underwent complete cytoreduction and perioperative chemotherapy (POC) develop recurrence [5]. 

This study is to analyze the prognostic factors and recurrence patterns of PM from colorectal cancer following the treatment with CRS + perioperative chemotherapy. 

## 2. Patients and Methods

### 2.1. Patients

From June 2004 to June 2012, 142 patients with PM of colorectal origin have been treated with CRS and perioperative chemotherapy. The patients were followed up until October 2012. PM was diagnosed by biopsy under laparotomy and laparoscopy or by the cytological examination of ascites.

Inclusion criteria were the following: (1) histopathologic or cytologic confirmation of PM; (2) performance status ≤2; (3) absence of hematogenous metastasis except liver metastasis of less than 3 and absence of remote lymph node metastasis; and (4) informed consent in accordance with the Japanese government guidelines of hospital. This study was approved by the ethical committee of Kishiwada Tokushukai Hospital.

### 2.2. Quantitative Evaluation of the Volume of PM and Assessment of Completeness of Cytoreduction

The tumor volume was quantified according to the Sugarbaker's peritoneal cancer index (PCI, range from 1 to 39) [4]. 

The entire small bowel and its mesentery are traced from the duodenojejunal flexure to the ileocecal junction. Both sides of the mesentery are inspected and palpated and evaluated the lesion size of the four zones of the small bowel (upper jejunum, lower jejunum, upper ileum, and lower ileum). The lesion size of each zone was counted and summed up as SB-PCI (range from 0 to 12). 

A completeness of cytoreduction score of CCR-0 (no residual visible tumor nodules) or CCR-1 (residual tumor nodules) was used to assess the completeness of cytoreduction. 

### 2.3. Methods of CRS and Hyperthermic Intraperitoneal Chemotherapy (HIPEC)

Complete cytoreduction was aimed at removing all macroscopic tumors with electrosurgical technique. The surgical resections consisted of a combination of peritonectomy procedures, described elsewhere [5]. Following surgery, 87 patients received HIPEC with mitomycin C at a dose of 20 mg/body and cisplatin 100 mg/body in 4 liter of saline warmed at 42 to 43 centigrade for 60 minutes. 

### 2.4. Evaluation of Pathologic Response to Preoperative Systemic Chemotherapy

Pathologic responses on PM were evaluated according to the general rules of gastric cancer treatment [5]. According to these rules, pathological response after chemotherapy is classified into 4 categories; Ef-0 reflects no pathologic response or response less than one-third of the tumor tissue, Ef-1 means that the cancer is detected in the tumor tissue ranging from one-third to less than two-thirds of the tumor tissue, and Ef-2 reflects the degeneration of cancer tissue in more than two-thirds of the tumor tissue, while Ef-3 responds to complete disappearance of the cancer cells.

### 2.5. Data Analysis

The survival analysis was performed using the Kaplan-Meier method and subgroups compared with a Log-rank test or Cox analysis. SPSS software version 11.5 (SPSS Inc., Chicago, USA) was used to analyze the data. Confidence intervals were taken at 95% levels, and *P* < 0.05 was considered significant. 

## 3. Results

### 3.1. Patients' Characteristics

Table 1 shows the clinicopathological characteristics of 142 patients. Of these, 68 patients were male, and 74 were female. The mean age of the patients was 54.1 years old (range from 21 to 76). 

Fifty-nine and 83 patients had synchronous and metachronous carcinomatosis, respectively. One-hundred ten patients have been treated with preoperative systemic chemotherapy (Table 1). Seventy-five patients had intraoperative PCI score less than 10, and 67 patients had PCI score above 11 (Table 2).

### 3.2. Surgical Procedures

Surgical procedures were performed in combination with peritonectomy and other organ resections according to involvement of entire abdomen and intra-abdominal organs in patients. Details of the procedures performed are listed in Table 2. 

Average bleeding volume and operation time were 1635 mL and 239 min, respectively. CCR-0 was achieved in 108 of 142 (76.1%) patients. Complete cytoreduction was also achieved in patients with higher PCI score above 11 (Table 2). Complete cytoreduction ratio was higher in patients with PCI score less than 10 (94.7%, 71/75) than those of PCI score above 11 (40.2%, 37/67). 

Regarding the correlation between SB-PCI score and CCR score, eighty-six of 94 (91.5%) patients with SB-PCI ≤ 2 and 22 of 48 (45.8%) patients with SB-PCI ≥ 3 received CCR-0 resection (*P* < 0.001). Causes of CCR-1 resection of 8 patients with SB-PCI ≤ 2 were liver metastasis in 2, emergency operation in 2, old age in 2, and sever local invasion in two patients.

HIPEC was done in 55 patients just after CRS.

### 3.3. Evaluation of Pathologic Response

Pathologic response of PM was evaluated in all patients. Eleven (7.9%) patients had a complete response (Ef-3), 13 (8.8%) patients had Ef-2 response, and 36 (25.7%) showed Ef-1 response. The other 82 (57.5%) had no response to preoperative systemic chemotherapy. 

### 3.4. Postoperative Complications

Among 142 patients, postoperative complications occurred in 61 patients (42.9%). Grade 1/Grade 2, Grade 3, and Grade 4 complications occurred in 36 (25.4%), 11 (7.7%), and 14 (9.9%), respectively. The overall operative mortality rate was 0.7% (1/142), and the cause of death was pulmonary embolism. Anastomotic leakages were the most frequent complication (*N* = 4). Bowel fistula, intraabdominal bleeding, abdominal abscess, rupture of right diaphragm, ileus, and wound dehiscence occurred in other cases.

### 3.5. Determination of Prognostic Parameters

**Figure 1:**
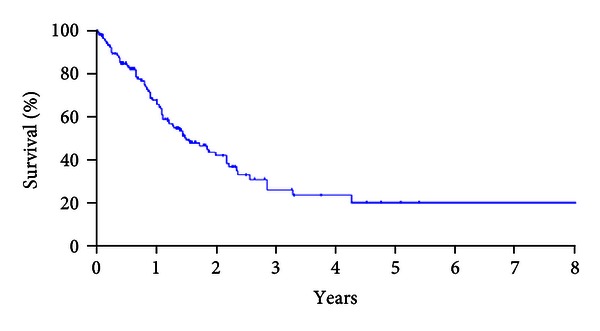
The overall survival of 142 patients, MST, and 5-year survival rate were 24.4 months and 23.4%.

**Figure 2:**
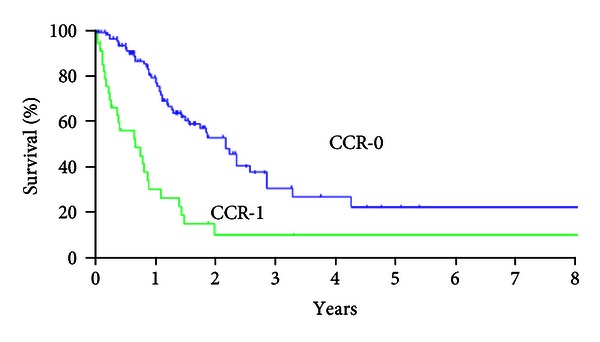
Survival curves of patients who underwent CCR-0 resection and CCR-1 resection (*χ* ^2^ = 26.791, *P* < 0.001). MST and 5-year survival rate were 25.9 months and 20% in patients who underwent to CCR-0 resection, but these in patients who underwent who CCR-1 resection were 8.0 months and 9.9%, respectively.

Figure 1 shows the overall survival of 142 patients, and the median survival time (MST) was 24.4 months.  Figure 2 shows the prolonged survival can be achieved in patients who underwent CCR-0 resection (*P* < 0.001). MST was 25.9 months in patients who underwent CCR-0 resection, but that in patients who underwent CCR-1 resection was 8.0 months. 

**Figure 3:**
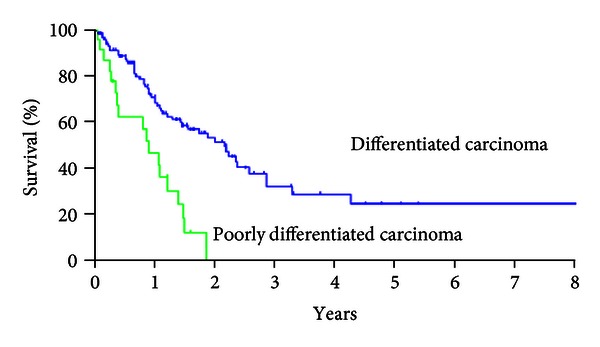
Survival curves of patients with differentiated and poorly differentiated carcinomas. MST and 5-year survival rate were 25.9 months and 24.6% in patients with differentiated carcinoma, but those in patients with poorly differentiated carcinoma were 10.7 months and 0% (*χ* ^2^ = 16.955, *P* < 0.001).

Patients with differentiated carcinoma had MST of 25.9 months, and that of patients with poorly differentiated carcinoma was 10.7 months (Figure 3, *P* < 0.001).

**Figure 4:**
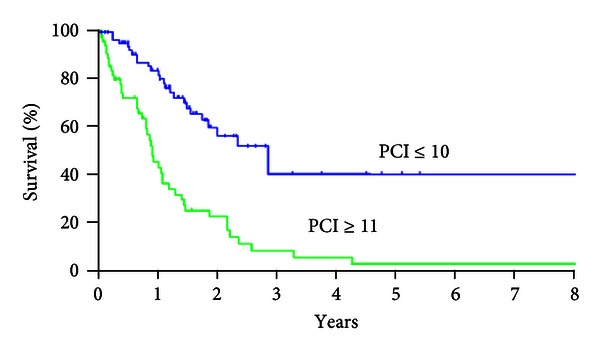
Survival curves of patients with PCI ≤ 10 and those with PCI ≥ 11. MST and 5-year survival rate in patients with PCI ≤ 10 were 33.7 months and 40.0%, but those in patients with PCI ≥ 11 were 10.5 months and 2.9%, respectively.

Figure 4 shows the survival curves of patients with PCI ≤ 10 and those with PCI ≥ 11. A significant survival difference was found between the two groups (*P* < 0.001).

**Figure 5:**
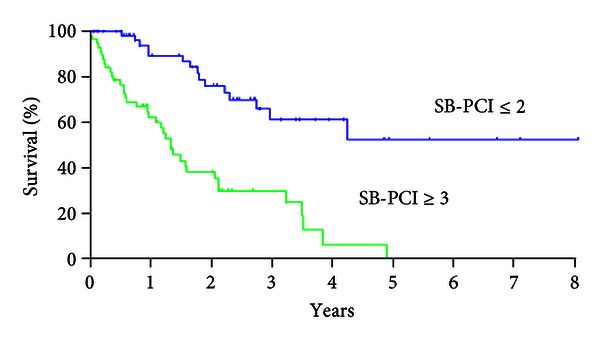
Survival curves of patients with small bowel PCI (SB-PCI) ≤2 and those with SB-PCI ≥ 3. MST and 5-year survival rate in patients with SB-PCI ≤ 2 were NR and 52%, but those in patients with SB-PCI ≥ 3 were 10.1 months and 0%, respectively.

Patients with small bowel PCI (SB-PCI) ≤2 had significantly better survival than those with SB-PCI ≥ 3 (Figure 5, *P* < 0.001). 

Log-rank test also demonstrated that HIPEC, age younger than 65 years old, and histologic response of EF-2/Ef-3 were significantly better prognostic factors (Table 3).

The Cox multivariate analysis demonstrated CCR-0 (*P* = 0.002), histologic type of differentiated carninoma (*P* = 0.008), PCI ≤ 10 (*P* < 0.001), and SB-PCI ≤ 2 (*P* = 0.027) were the independent good prognostic factors (Table 3).

## 4. Discussion

The recent therapeutic approach for colorectal cancer patients with PM is a comprehensive treatment consisting of CRS plus peroperative intraperitoneal or systemic chemotherapy. Macroscopic peritoneal nodules are removed with peritonectomy techniques, and the residual micrometastases are eradicated with HIPEC. 

In advanced colorectal cancer, median survival time is one to two years with palliative systemic chemotherapy [6]. Sanoff et al. [6] studied 1691 patients, who were randomly assigned to one of seven fluorouracil-, oxaliplatin-, and irinotecan-containing regimens. The observed 5-year survival with infusional fluorouracil, leucovorin, and oxaliplatin (FOLFOX) was 9.8% that was better than with irinotecan plus bolus fluorouracil and leucovorin (IFL; 3.7%) or with bolus irinotecan/oxaliplatin (IROX; 5.1%). This study suggests that, in terms of OS, TTP, response rate, and toxicities were concerned FOLFOX which was superior to IFL or IROX regimens. 

More recently, Franko et al. evaluated outcomes of patients with peritoneal carcinomatosis from colorectal cancer (pcCRC) enrolled onto two prospective randomized trials of chemotherapy and contested that with other manifestations of metastatic colorectal cancer (non-pcCRC) [7]. As a result, pcCRC is associated with a significantly shorter OS and PFS as compared with other manifestations of mCRC, and patients with pcCRC had higher risk of death owing to all causes rather than patients without pcCRC. 

On the other hand, the combination of systemic chemotherapy after aggressive cytoreduction surgery with concurrent intraperitoneal chemotherapy has demonstrated a remarkable improvement in survival. The median survival was 22.3 months in 562 advanced colorectal cancer patients treated with CRS plus HIPEC, and systemic chemotherapy [8]. Five-year survival after this comprehensive treatment was reported to be 45% that was higher than that of chemotherapy alone [9]. Multivariate analyses showed that completeness of cytoreductive surgery, tumor burden less than the threshold values, histological differentiation, HIPEC and systemic chemotherapy have been reported as the independent prognostic indicators for prolonged survival [3, 9]. Among these prognostic factors, completeness of cytoreduction (CCR-0) was found to be the most important prognostic indicator. The five-year survival rate in patients who underwent CCR-0 was 45%, but in patients with incomplete CRS (CCR-1) was only 5% [9]. 

Nearly all authors are agreeing with that the complete cytoreduction (CCR-0) is the essential prognostic indicator for survival [9, 10]. The 5-year survival rates in patients who had a CCR-0 resection ranged from 22% to 49% [9–11]. Accordingly, CCR-0 is a preferable to achieve the long-term survival in these patients [12]. 

The present study also demonstrated that MST of CCR-0 group was significantly longer than that of CCR-1 group. In addition, Verwaal [9] said the median survival time was also shorter in the standard systemic chemotherapy arm compared to the patients treated with CRS plus HIPEC. Therefore, CRS plus HIPEC is now considered as a gold-standard of the treatment of PM which originated from colorectal cancer. 

It has been reported that tumor differentiation was the prognostic factor in these patients, and almost all of the patients with poorly differentiated carcinoma died because of recurrence within 4 years even after CCR-0 resection [8, 13–15]. The present study also demonstrated that the patients with differentiated carcinoma had a significantly better survival than that of patients with poorly differentiated carcinoma. All the patients died of intra-abdominal recurrence within 2 years after CRS. 

PCI deserves special attention because it objectively quantifies the tumor burden and closely correlated with the rate of complete cytoreduction and survival. The low cut-off level of PCI associated with a favorable survival has been reported. Sugabaker et al. [16] reported that MST for PCI < 20 was 41 months, compared with 16 months for PCI > 20. Elias et al. [11] reported that PCI > 15 was the threshold level for significant poor prognosis. Yan and Morris [17] found the PCI ≤ 10 was a significant favorable prognostic factor. In the present study, survival of patients with PCI ≤ 10 was significantly better than that of patients with PCI ≥ 11, and 5-year survival rate of patients with PCI ≤ 10 was 40.0%, which was significantly better than that of patients with PCI ≥ 11 was 2.9%.

Involvement of the small bowel and its mesentery is the most important limiting factor for CCR-0 resection. Diffusion of small bowel involvement is the most frequent cause of CCR-1 resection. Since PM from colorectal cancer often invades the marginal arteries of the small bowel, where the terminal arteries enter the bowel wall, whole layer of bowel wall must be inevitably removed to achieve CCR-0 resection. After the meticulous check of the entire small bowel, a decision should be done to perform bowel resection while leaving adequate bowel length for the normal nutritional function and minimizing the number of anastomoses. Esquival et al. reported that there is no surgical option to remove all affected sites of small bowel, if even evidence of intestinal obstruction at more than one site [18]. So far, no report has been published about the correlation between survival and the SB-PCI scores. The present study demonstrated that the CCR-0 resection rate in patients with SB-PCI ≤ 2 was significantly higher (91.5% 86/94) than that in patients with SB-PCI ≥ 3 (45.8%, 22/48). In addition, Cox multivariate analysis demonstrated that SB-PCI ≤ 2 was an independent good prognostic factor. Despite a high frequency of small bowel resection (58.4%, 83/142) to perform CCR-0 resection in this study, all the patients with SB-PCI ≥ 3 died of recurrence within 4 years after the comprehensive treatment. Accordingly, the threshold of the SB-PCI must be ≤2 for colorectal cancer. In colorectal cancer patients, an extended removal of small bowel will cause not only a short bowel syndrome, but also a recurrence within short time due to the aggressive biological behavior. Recently, neoadjuvant intraperitoneal/systemic chemotherapy (NIPS) was reported to reduce the SB-PCI scores, and to improve the postoperative survival in gastric cancer [19]. These results strongly indicated that neoadjuvant chemotherapy may increase the CCR-0 resection rate by eradicating PM on small bowel before CRS. In particular, patients who are diagnosed as SB-PCI ≥ 3 by preoperative image diagnoses or laparotomy/laparoscopy should be treated with neoadjuvant chemotherapy or laparoscopic HIPEC. 

In gastric cancer with PM, pathologic response after neoadjuvant chemotherapy was an independent prognostic factor [19]. Neoadjuvant chemotherapy induces stage migration, eradication of micrometastasis outside the surgical field and the improvement of CCR-0 resection ratio. The present study demonstrated that Log-rank test showed a significant better survival in patients with Ef-2 or Ef-3 response than those with Ef-1 or Ef-0 response. However, by a multivariate analysis, histological response after neoadjuvant chemotherapy did not emerge as an independent prognostic factor. In gastric cancer, histologic responders after NIPS were experienced in more than 50% of patients [19]. In the present study, however, only 16.7% of patients showed Ef-3 or Ef-2 response after systemic chemotherapy for colorectal cancer patients. In colorectal cancer with PM, low response rate after preoperative systemic chemotherapy may be the cause of this factor as a nonindependent prognostic factor. 

Accordingly, further studies need to be conducted with new perioperative chemotherapeutic approaches combined with molecular targeting therapies. 

## Figures and Tables

**Table 1:** Patients and tumor characteristics.

Characteristics	
Gender	
Male	68
Female	74
Tumor location	
Colon	6
Rectum	136
Lymph node status	
N0	50
N1	64
N2, N3	28
Diagnosis of carcinomatosis	
Synchronous carcinomatosis	59
Metachronous carcinomatosis	83
Systemic chemotherapy prior to cytoreduction	
None	32
FOLFOX (+bevacizumab; BV)	60 (24)
FOLFIRI (+BV or +cetuximab)	17 (2, 5)
Xeloda (+oxaliplatin or +BV)	17 (14, 5)
S1 (+paclitaxel)	10 (5)
Others (IRIS^#^, TJ^$^, SOX^&^, and HIPEC^¥^)	6

^#^IRIS: Irinotecan +S1, ^$^TJ: taxol + CBDCA.

^&^SOX: S1+ Oxaliplatin.

^¥^HIPEC: hyperthermic intraperitoneal chemotherapy.

**Table 2:** Operation methods.

Completeness of cytoreduction (CCR)		
CCR-0 (complete cytoreduction)	108	
CCR-1 (incomplete cytoreduction)	34	
CCR-0 resection ratio and PCI levels^#^		
PCI ≤ 10	71/75 (94.7%)	*P* = 0.015
11 ≤ PCI ≤ 20	23/31 (74.2%)	
21 ≤ PCI ≤ 30	14/26 (53.8%)	
31 ≤ PCI ≤ 39	0/10 (0%)	
CCR-0 resection ratio and SB-PCI levels^#^		
SB-PCI ≤ 2	86/94 (91.5%)	*P* < 0.001
SB-PCI ≥ 3	22/48 (45.8%)	
CCR-0 resection ratio and histologic type		
Differentiated type	97/119 (81.5%)	*P* = 0.015
Poorly differentiated	11/23 (43.4%)	
Removed peritoneal zones and organs		
Left diaphragmatic peritoneum	60	
Right diaphragmatic peritoneum	61	
Pelvic peritoneum	99	
Splenectomy	131	
Gastrectomy		
Distal gastrectomy	8	
Wedge resection	4	
Cholecystectomy	140	
Small bowel resection	83	
Large bowel resection		
Ileocaecal resection	2	
Total colectomy	7	
Right hemicolectomy	21	
Left hemicolectomy	8	
Sigmoidectomy	1	
Rectosigmoidectomy	28	
Resection of anastomosis	36	
Amputation of rectum	4	
Bleeding volume (mean ± SE)	1635 ± 1234 mL	
Operation time (mean ± SE)	239 ± 103 min.	
No. of resected peritoneal zones	5.1 ± 3.2 (1–13)	
No. of resected organs	3.8 ± 2.0 (0–8)	
HIPEC		
Not done	55	
done	87	

^#^SB-PCI: peritoneal cancer index on the small bowel.

**Table 3:** Results of multivariate analyses and Log-rank test. The Cox multivariate analysis demonstrated CCR-0 (*P* = 0.002): histologic type of differentiated carcinoma (*P* = 0.008), PCI ≤ 10 (*P* < 0.001), and SB-PCIl ≤ 2 (*P* = 0.027) were the independent good prognostic factors.

Variables	Cox hazard model				Log-rank test	
	*P* value	Relative risk	95% confidential interval	Hazard ratio	*χ* ^2^	*P* value

Sex						
Male versus female	0.303	1.423	0.727–2.724	1.349	2.612	0.106
HIPEC						
Done versus not done	0.072	0.555	0.292–1.054	4.446	5.501	0.019
CCR						
CCR = 0 versus CCR = 1	0.002	3.47	1.563–7.703	11.707	26.729	<0.001
Age						
65 y.o. < versus 65 y.o. ≤	0.141	1.702	0.837–3.461	1.165	3.903	0.005
Lymph node						
N0 versus N+	0.562	1.208	0.639–2.280		0.693	0.406
PCI				0.974		
PCI = 1 < 0 versus PCI ≥ 11	0.001	3.43	1.925–3.232	8.608	22.564	<0.001
Histology						
Differentiated versus Poorly	0.008	2.77	1.306–5.872	5.288	16.95	<0.001
Histologic effect after chemotherapy						
EF = 0,1 versus EF = 2,3	0.101	0.413	0.143–1.188	3.501	6.856	0.003
SB-PCI						
SB-PCI ≥ 3 versus SB-PCI ≤ 2	0.027	3.356	1.145–9.238	6.182	27.25	<−0.001
